# Personal

**Published:** 1908-11

**Authors:** 


### PERSONAL.

---

DR. JOHN F. ERDMANN, of New York, at a meeting of the Board of Directors of the New York Post-Graduate Medical School and Hospital, held October 7, 1908, was appointed Professor of Surgery for the ensuing year.

---

DR. GEORGE M. GOULD, founder and formerly editor of *American Medicine,* has removed from Philadelphia, to Ithaca, N. Y., where his offices are established at Cayuga Heights.

---

MR. FRANK A. RUF, of Saint Louis, has been created a Knight of the Order of the Lion and the Sun by the Shah of Persia. Mr. Ruf has been a collector of Persian textile art treasures for years, and this coming to the knowledge of the Shah, an order was issued—an Imperial firmin—commanding the vice-consul at Saint Louis, to confer the decoration which was done recently with ceremony.

